# The association between community-associated *Staphylococcus aureus* colonization and disease: a meta-analysis

**DOI:** 10.1186/s12879-018-2990-3

**Published:** 2018-02-21

**Authors:** Marina W. Kim, Ben K. Greenfield, Robert E. Snyder, Craig M. Steinmaus, Lee W. Riley

**Affiliations:** 1 School of Public Health, University of California, Berkeley, CA USA; 2 Department of Environmental Sciences, Southern Illinois University, Edwardsville, IL USA

**Keywords:** *Staphylococcus aureus*, Infection, Colonization, Community-associated

## Abstract

**Background:**

Colonization with *Staphylococcus aureus* is a well-defined risk factor for disease in hospitals, which can range from minor skin infections to severe, systemic diseases. However, the generalizability of this finding has not been thoroughly investigated outside of the hospital environment. We aimed to assess the role of *S. aureus* colonization as a risk factor for disease in the community.

**Methods:**

We performed a meta-analysis of observational studies and searched PubMed for articles published between December 1979 and May 23, 2016. We included cohort, cross-sectional, and case-control studies that reported quantitative estimates of both *S. aureus* colonization and disease statuses of all study subjects. We excluded studies on recently hospitalized subjects, long-term care facilities, surgery patients, dialysis patients, hospital staff, *S. aureus* outbreaks, and livestock-associated infections. Our meta-analysis was performed using random-effects analysis to obtain pooled odds ratios (ORs) to compare the odds of *S. aureus* disease with respect to *S. aureus* colonization status.

**Results:**

We identified 3477 citations, of which 12 articles on 6998 subjects met the eligibility criteria. Overall, subjects colonized with *S. aureus* were more likely to progress to disease than those who were non-colonized: (OR 1.87, 95% CI 1.21–2.88, *n* = 7 studies). We observed a larger effect with methicillin-resistant *S. aureus* colonization (7.06, 4.60–10.84, *n* = 7 studies). However, the methicillin-sensitive *S. aureus* colonization was not associated with greater odds of disease (1.20, 0.69–2.06, *n* = 4 studies). Heterogeneity was present across studies in all of the subgroups: *S. aureus* (I^2^ = 95.0%, *χ*^2^ = 120.3, *p* < 0.001), MRSA (I^2^ = 92.8%, *χ*^2^ = 82.8, *p* = *p* < 0.001), and MSSA (I^2^ = 86.3%, *χ*^2^ = 21.8, *p* < 0.001).

**Conclusions:**

While the majority of papers individually support the assumption that colonization is a risk factor for *S. aureus* disease in the general population, there is marked heterogeneity between studies and further investigation is needed to identify the major sources of this variance. There is a shortage of literature addressing this topic in the community setting and a need for further research on colonization as a focus for disease prevention.

**Electronic supplementary material:**

The online version of this article (10.1186/s12879-018-2990-3) contains supplementary material, which is available to authorized users.

## Background

*Staphylococcus aureus* is an important bacterial pathogen that can cause opportunistic disease in both community and hospital settings. While *S. aureus* disease has been associated with exposures in hospital environments, many people without any previous history of hospitalization have also developed disease outside of traditional health care settings since the 1990s [1]. Community-associated methicillin-resistant *S. aureus* (CA-MRSA) strains have also gradually circulated in health care settings, demonstrating that some clones are not necessarily more frequently found in the original setting where they were first described (i.e., healthcare associated [HA-MRSA] v. CA-MRSA) [2]. Clinical manifestations of community-associated *S. aureus* disease can include skin and soft tissue infections (SSTI) [3]. It is also an important cause of bacteremia, infective endocarditis, pneumonia, bone and joint infections, and other potentially serious diseases, that vary in prevalence depending on geographic area and the socio-demographic characteristics of the populations [4].

In general, symptomatic *S. aureus* infections begin when the skin or mucosal barrier is broken and the normally commensal bacteria reaches the bloodstream or elsewhere, especially among those asymptomatically carrying *S. aureus* [5]. The nasal epithelium is the most common location for *S. aureus* colonization, while carriage on the perineum, axilla, and other skin sites occur less frequently [6, 7]. A recent worldwide review of *S. aureus* nasal carriage estimated that the average prevalence of nasal colonization in the general population is 24%, with the highest proportions of persons colonized with methicillin-resistant *S. aureus* (MRSA) and methicillin-susceptible *S. aureus* (MSSA) residing in North and South America, respectively [8]. Bacterial pathogenesis is not fully understood, although virulence determinants of particular strains of *S. aureus* may play a role [5]. The multifactorial nature of staphylococcal carriage, pathogenesis, and host vulnerability has made it difficult to predict the risk of progression to disease from a colonized state.

The relationship between *S. aureus* colonization and disease has been widely confirmed in healthcare settings. Several systematic reviews have reported that colonization with *S. aureus* is associated with an increased risk of disease with the organism in hospital patients who undergo surgery, hemodialysis, peritoneal dialysis, or intensive care [9–13]. However, this relationship has not been well-described among those without previous exposure to a healthcare setting. More than 3400 studies of *S. aureus* colonization and disease have been done, but few evaluate the incidence or prevalence of both community-associated colonization and disease on the same individual. If the association between colonization and disease is more adequately investigated in communities as it has been in hospitals, we may gain a better understanding of whether the potential risk of progression to disease in the general population is different when compared to hospitalized populations. This information is important to properly inform disease prevention strategies in the community settings. We performed a meta-analysis to evaluate the strength of the common assumption that *S. aureus* colonization is a risk factor for symptomatic *S. aureus* infection in the community setting.

## Methods

### Search strategy and selection criteria

We conducted this meta-analysis in accordance with the Preferred Reporting Items for Systematic Reviews and Meta-Analyses (PRISMA) guidelines [14]. We searched PubMed to identify papers that reported the prevalence of asymptomatic, community-associated *S. aureus* colonization and the incidence or prevalence of community-associated *S. aureus* disease. Each literature search consisted of a variation on the following three categories of search terms: (1) pathogen description, (2) exposure, and (3) outcome (Additional file 1). We used these categories as a structure for various combinations of key terms, expressed as any possible combination of the following: (1) “*Staphylococcus aureus,*” or “methicillin-resistant *Staphylococcus aureus,*” or “MRSA,” or “methicillin-susceptible *Staphylococcus aureus,*” or “MSSA,” and (2) also including “colonization,” or “carriage” and (3) “infection”.

**Fig. 1:**
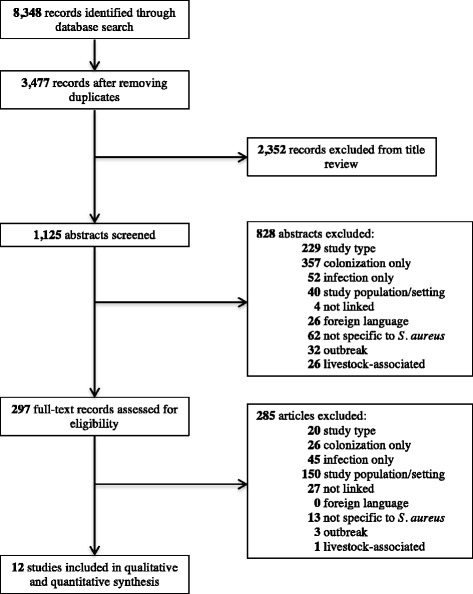
Study selection. Study type = reviews, editorials, commentaries, and other non-epidemiological studies. Study population/setting = studies that included participants or settings that did not match the eligibility criteria. Not linked = joint distributions not given for relative risk calculation

Our literature searches did not place restrictions on publication date, capturing articles published between December 1979 and May 23, 2016, the date of the most recent literature search. We included prospective cohort, retrospective cohort, cross-sectional, and case-control studies that reported quantitative estimates of both the *S. aureus* colonization and disease statuses of all study subjects. Only articles written in English, Spanish, French, or Portuguese were reviewed. To focus the study on *S. aureus* of community origin, we excluded articles with subjects who underwent surgery or were hospitalized for more than 48 h within one year prior to the start of the study, patients who were concurrently hospitalized, residents of long-term health care facilities, peritoneal or hemodialysis patients, hospital staff, or individuals who have regular contact with livestock (Fig. 1). Additionally, we excluded outbreak studies to minimize the possibility that skin-to-skin transmission or fomite contact led to transmission and/or disease [15].

### Data analysis

One researcher (MWK) reviewed the titles and abstracts of all articles obtained through the literature review process to select studies that qualified for full-text review according to the inclusion and exclusion criteria, in consultation with other authors (BG, RS, CS, LR). In the process of full-text review, the following items were obtained from each study and systematically recorded on a spreadsheet: year of publication, study design, study setting, geographical area, study period, duration of follow-up (if applicable), colonization isolate collection site, method of disease diagnosis, *S. aureus* type, anatomical site of disease, study population size, and study subject criteria. *S. aureus* type was described in three categories, based on how it was reported in the original article: (1) all *S. aureus*, (2) MRSA, and (3) MSSA. Some participants in the MRSA or MSSA categories may also be found in the *S. aureus* category, but there were no overlaps of cohorts within each of the summary measurements. We assessed the proportion of subjects who were colonized, non-colonized, symptomatically infected, and not symptomatically infected with *S. aureus.* If joint distributions of colonization and disease could not be ascertained directly from an article, we contacted the authors to inquire about the availability of those data.

Our primary outcome measure was the relative risk of symptomatic *S. aureus* infection comparing colonized individuals versus non-colonized individuals. We performed separate analyses for general *S. aureus,* as well as specific types of *S. aureus* by methicillin susceptibility: MRSA and MSSA. We calculated summary ORs and 95% confidence intervals for each subgroup using an inverse variance weighted random-effects model, employing the chi-square test of homogeneity test statistic and an alpha of 0.05 [16, 17]. We also evaluated consistency of study results with the I^2^ statistic, which represents the percentage of variation across the studies that is not due to chance [18]. Some studies reported data on multiple *S. aureus* types and were included in more than one subgroup analysis. In a secondary analysis, we calculated pooled ORs stratified by HIV infection to evaluate whether or not risk of *S. aureus* disease varied by immune status. We assessed the presence of publication bias in each subgroup by creating funnel plots displaying effect size vs. variance [19]. We further evaluated risk of publication bias with Egger’s test [20]. We conducted a sensitivity analysis to determine whether some studies disproportionately affected the overall summary ORs. Data assembly and statistical analyses were performed in Microsoft Excel and STATA V 13.1 (StataCorp. 2013. *Stata Statistical Software: Release 13*. College Station, TX: StataCorp LP).

## Results

Our literature search identified 3477 unique articles, of which 297 (8.5%) were eligible for full-text evaluation. Of 297 articles included in our full-text review, 285 (96.0% of those eligible) did not satisfy eligibility criteria for inclusion in analysis. Of the articles excluded from full text review, 98 (34.4%) had insufficient data to calculate relative risks of colonization versus disease status and 150 (52.6%) did not meet the inclusion criteria based on study setting or population (Fig. 1). Finally, 12 studies with a total of 6998 study subjects met our inclusion criteria for further analysis [21–32]. These studies consisted of seven prospective cohort studies [21, 23–25, 29, 30, 32], two case-control studies [26, 27], and three cross-sectional studies published between 1999 and 2015 (Additional file 2) [22, 28, 31]. Study subjects included 1289 HIV-infected individuals, 552 adult outpatients, 687 pediatric outpatients, 3066 military trainees, 490 prison inmates, and 914 other adults and children in the community. Only two of the 12 studies were done outside of the United States: Taiwan and Italy [28, 31].

**Fig. 2:**
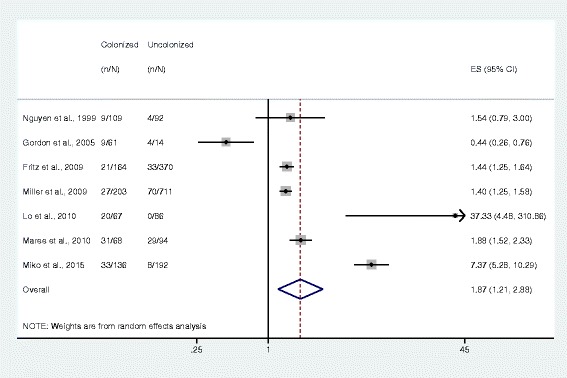
Forest plot of random-effects meta-analysis comparing the odds of *S. aureus* infection among colonized and uncolonized individuals. ES = effect size. The gray shaded boxes vary in size according to the weight given to each study

**Fig. 3:**
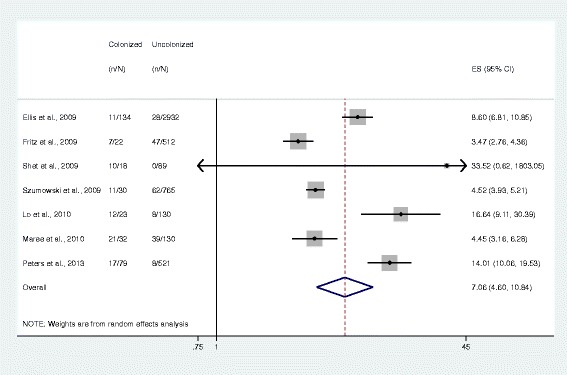
Forest plot of random-effects meta-analysis comparing the odds of methicillin-resistant *S. aureus* infection among colonized and non-colonized individuals. ES = effect size. The gray shaded boxes vary in size according to the weight of each study

**Fig. 4:**
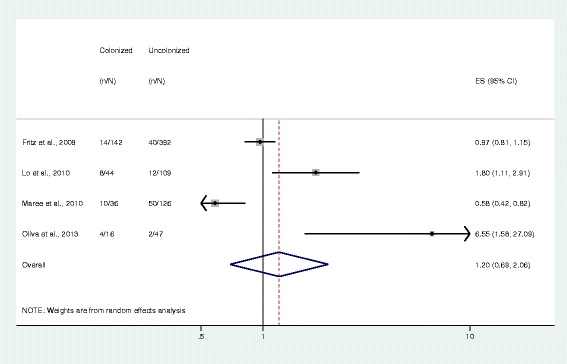
Forest plot of random-effects meta-analysis comparing the odds of methicillin-susceptible *S. aureus* infection among colonized and non-colonized individuals. ES = effect size. The gray shaded boxes vary in size according to the weight of each study

Meta-analysis results showed that subjects colonized with *S. aureus* were more likely to have *S. aureus*-related disease than those who were non-colonized (OR 1.87, 95% CI 1.21–2.88, *n* = 7 studies; Fig. 2), with a *S. aureus* colonization prevalence of 34.1% among 2367 subjects. MRSA colonization was associated with greater odds of having disease (7.06, 4.60–10.84, *n* = 7 studies) compared to MSSA colonization, which was not statistically significant (1.20, 0.69–2.06, *n* = 4 studies). The prevalence of MRSA colonization was 6.2% among 5417 subjects and the prevalence of MSSA colonization was 26.1% among 912 subjects (Figs. 3 and 4).

**Fig. 5:**
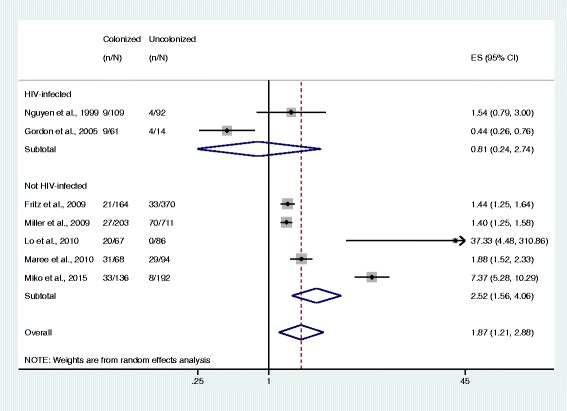
Forest plots of random-effects meta-analysis comparing the odds of *S. aureus* infection among colonized and non-colonized individuals, stratified by HIV infection status. ES = effect size. The gray shaded boxes vary in size according to the weight of each study

**Fig. 6:**
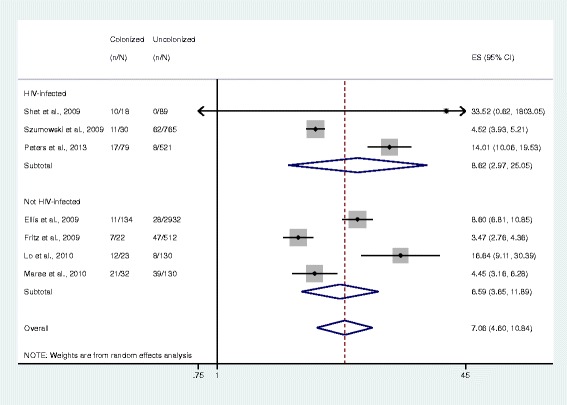
Forest plots of random-effects meta-analysis comparing the odds of methicillin-resistant *S. aureus* infection among colonized and non-colonized individuals, stratified by HIV infection status. ES = effect size. The gray shaded boxes vary in size according to the weight of each study

The pooled ORs comparing the odds of disease and colonization status were somewhat lower in a subgroup of HIV-infected study participants (OR 0.81, 95% CI 0.24–2.74, *n* = 2 studies) compared to HIV-uninfected participants (2.52, 1.56–4.06, *n* = 5 studies). However, this difference is not statistically significant and does not provide sufficient evidence for effect measure modification according to previously described methods for comparing two measures of association (Fig. 5) [33]. For studies in the MRSA subgroup, the pooled ORs comparing the odds of disease and colonization status among HIV-infected study participants (8.62, 2.97–25.05, *n* = 3 studies) and HIV-uninfected populations (6.59, 3.65–11.89, *n* = 4 studies) were also not significantly different (Fig. 6).

The tests of homogeneity indicated that heterogeneity existed for all subgroups: *S. aureus* (I^2^ = 95.0%, *χ*^2^ = 120.3, *p* < 0.001), MRSA (I^2^ = 92.8%, *χ*^2^ = 82.8, *p* < 0.001), and MSSA (I^2^ = 86.3%, *χ*^2^ = 21.8, *p* < 0.001). In our assessment of publication bias, Egger’s test did not indicate a significant risk of bias due to the small study effect among studies in each subgroup analysis for *S. aureus* (bias = 2.26, *p* = 0.48), MRSA (bias = 3.30, *p* = 0.26), or MSSA (bias = 2.20, *p* = 0.49). However, the funnel plots appeared asymmetrical and seemed to display contradictory results (see Additional files 3, 4 and 5). It is possible that publication bias may exist, but our analysis contained too few studies in each subgroup (< 10); thus the Egger’s test and funnel plots cannot confidently assert whether or not publication bias was present. While we did not observe a clear pattern consistent with publication bias, it is important to continue to publish null results in light of the lack of studies available on this subject.

Additionally, we observed several variations in reporting both exposure and outcome. All studies collected nasal swabs from subjects to test for the presence of *S. aureus* carriage by culture, PCR, or both. Eight studies obtained specimens solely from the nasal cavity, whereas the remaining four studies also tested an additional anatomic site for colonization, including the perineum, axilla, or oropharynx. The method for evaluating disease also differed across studies. Six of the 12 studies relied on laboratory confirmation of disease from clinical isolates, four determined the presence of disease by physician diagnosis without laboratory confirmation, one study collected outcome information from self-reported episodes of disease with medical chart review for a proportion of subjects [24], and one study determined disease by self-report only [22]. Additionally, seven studies assessed molecular concordance between colonizing and infecting isolates by comparing pulse-field type, sequence type, or *spa* type. These studies estimated that the percentage of *S. aureus* strains that were concordant ranged from 25 to 100%, but five of the seven studies reported that some isolates were not available for typing (Table 1).

**Table 1:** Concordance of *Staphylococcus aureus* molecular typing between colonizing and infecting isolates

Article	N	Infected and colonized (N)	Concordant strains (N, %)^a^	Isolate pairs typed (N, %)^b^	Molecular typing method
Nguyen et al., 1999 [32]	201	9	6 (86%)	7 (78%)	PFGE
Gordon et al., 2005 [30]	75	9	2 (25%)	8 (89%)	PFGE
Ellis et al., 2009 [21]	3066	11	11 (100%)	11 (100%)	PFGE
Shet et al., 2009 [29]	107	10	4 (100%)	4 (40%)	PFGE
Maree et al., 2010 [27]	162	21	14 (93%)	15 (71%)	PFGE
Peters et al., 2013 [25]	600	17	14 (100%)	14 (82%)	PFGE
Miko et al., 2015 [26]	328	33	22 (71%)	31 (94%)	*spa* typing

*PFGE* pulse-field gel electrophoresis

^a^Percentage of concordant strains = (# concordant strains)/(# of isolate pairs typed)

^b^Percentage isolate pairs typed = (# of isolate pairs typed)/(# infected and colonized)

A sensitivity analysis excluding the two articles in which patients self-reported episodes of skin infections [22, 24] during the study period yielded the following results: *S. aureus* (OR 2.52, 95% CI 0.93–6.83, *n* = 5 studies), MRSA (8.23, 5.06–13.38, *n* = 6 studies), and MSSA (1.59, 0.53–4.78, *n* = 3 studies). The removal of these studies among all subgroup analyses slightly increased the effect sizes, but did not have a disproportionate influence on the overall conclusions for the subgroups.

## Discussion

Despite the widespread concern about CA-MRSA [1], this is, to our knowledge, the first comprehensive and systematic evaluation of the risk of *S. aureus* disease from colonization focused on community-associated infection. While the results of this meta-analysis suggest that the epidemiology of *S. aureus* may differ across *S. aureus* types according to their antibiotic susceptibility profiles, it is important to note that we have observed high levels of heterogeneity in this meta-analysis and any interpretation of these results is limited. Still, the majority of the relative measures of association from individual studies were found to be above the null, and supports the overall positive associations observed in this study. We found that individuals colonized with MRSA had higher odds of disease compared to those who were not colonized with MRSA, and seven of seven study effect sizes in this meta-analysis were above the null value despite the presence of heterogeneity. We also observed the same relationship for persons colonized with any kind of *S. aureus*. The effect sizes included in this subgroup analysis ranged from OR = 0.44 to OR = 37.33 and six of seven studies yielded effect sizes that were both statistically significant and above the null. We did not find conclusive evidence of this when examining MSSA alone, and the two studies that examined both MSSA and MRSA both exhibited lower ORs for MSSA than for MRSA [24, 28]. Still, MRSA strains are not necessarily more virulent than MSSA strains; genetic factors, which may also be associated with resistance status, likely play a more substantial role in bacterial virulence [34].

In previous studies estimating disease incidence only, HIV-positive patients had a higher risk of CA-MRSA disease than the general population [35, 36]. Additionally, a longitudinal study using a series of cultures to test for colonization over time reported that a larger proportion of HIV-infected individuals persistently carried *S. aureus* compared to HIV-uninfected individuals [37]. Despite these data suggesting that HIV infection may be associated with higher prevalence of colonization and incidence of disease, our findings suggest that the relationship between colonization and disease in the community does not change with different statuses of HIV infection. HIV-infected individuals were not significantly more likely to be symptomatically infected with *S. aureus* or MRSA than HIV-uninfected individuals if they had also been colonized with *S. aureus* or MRSA (*p* > 0.05). This finding is unexpected, as HIV is universally reported to increase the risk of most infectious diseases due to its immunosuppressive effect. It is also important to note that the number of studies representing HIV-infected participants was small for each subgroup: two for *S. aureus*, three for MRSA, and one for MSSA. More data may be useful to better evaluate the role of immune status in the progression to *S. aureus* disease.

There are several limitations to this meta-analysis and the studies included in the present analysis, drawing attention to the variation in research practices and definitions across these studies. First, three studies were cross-sectional, thus making it difficult to establish a causal relationship between colonization and disease without confirming that colonization preceded disease. When study designs limit the ability to establish a temporal relationship between colonization and disease, genotyping can detect identical colonizing and infecting staphylococcal isolates, which is commonly used an indicator for endogenous disease [38, 39]. However, none of the three cross-sectional studies included in this meta-analysis compared the strain types of colonizing and infecting isolates. Consequently, we were unable to ascertain whether colonization occurred before or after disease onset. Furthermore, it was not possible to implicate *S. aureus* as the disease-causing pathogen in six of 12 studies that did not rely on laboratory confirmation of clinical isolates.

Second, methods for evaluating *S. aureus* colonization varied across studies. Longitudinal studies on nasal colonization have described three types of carriage patterns: persistent, intermittent, and non-carriage, which can only be determined by collecting multiple samples over time [8]. Only four of 12 studies consistently collected more than one swab per individual at different times within the study period. Distinguishing between persistent and intermittent nasal carriage may be important because persistent nasal carriers have been found to have an increased bacterial load with studies suggesting that high bacterial loads increase risk of disease [40]. Moreover, we observed differences in staphylococcal carriage between different anatomical sites across studies. Only four of 12 studies tested more than one area. While the anterior nares is the primary site of colonization, several population studies have found that a significant number of individuals carry *S. aureus* only in the throat [41–44]. Thus, throat culture in addition to nares culture may help detect more colonized persons and can be particularly useful when evaluating the risk of *S. aureus*-related respiratory infections. Children and HIV-infected adults also have an especially high prevalence of carriage in the perineum [45]. Studies that assess colonization only once or at only one anatomical site may lack the sensitivity for accurately categorizing colonization status in study subjects.

Third, the method for diagnosing disease also varied across studies. As discussed, the gold standard for establishing *S. aureus* as a cause of disease is laboratory culture or PCR of the clinical isolate, supplemented by genotyping. The cause of disease was less clear in the six studies that relied exclusively on physician diagnosis or self-report in the absence of laboratory confirmation. For two studies that used self-report as a method to determine disease, participation in a study in which the investigators periodically request information about recent skin infections could potentially lead to the over-reporting of symptomatic infection episodes due to subjects’ heightened awareness. While culture-confirmed disease is the most reliable way to diagnose staphylococcal disease, one limitation of community-based studies is that it is more challenging and costly to administer laboratory tests compared with hospital-based studies in which these laboratory services are readily available and clinically necessary for patient care.

Of the more than 3000 articles published on colonization and symptomatic infection of *S. aureus*, only 12 met the inclusion criteria for review and inclusion in our meta-analysis. This highlights the paucity of scientific literature describing the risk of community-onset *S. aureus* disease in populations without hospital-associated risk factors. We excluded 68 of 135 studies conducted in eligible study populations during the full-text review process due to a lack of complete data to calculate relative measures of association. This may indicate a lack of systematic data collection on colonization status versus disease onset in the same cohort. Future studies seeking to compare the risk of disease between colonized and uncolonized individuals might consider reporting both disease and carriage statuses of all study subjects. Nevertheless, as shown in this meta-analysis, the existing literature suggests that the relationship between colonization and disease still exists apart from traditional hospital-related risk factors.

Given the high prevalence of colonization in the general population (~ 24%) [8], and the role that colonized individuals play in *S. aureus* transmission [1, 46], colonization status should be considered when establishing public health practices to prevent *S. aureus* disease especially among high-risk regions and vulnerable subpopulations. Nasal mupirocin treatment is an effective remedy under the assumption that decreasing *S. aureus* carriage can directly reduce the risk of symptomatic infection. Notably, this method has a lower risk for contributing to the development of antibiotic resistance compared to orally administered antibiotics [47]. However, randomized controlled trials among both United States military trainees and HIV-infected adults have revealed that decolonization by mupirocin made no difference in the incidence of symptomatic infections, and did not did prevent future *S. aureus* colonization [48]. Even with treatment, infection management, hygiene, and other methods for reducing colonization, preventing disease can sometimes be an ongoing effort.

A study conducted in 2000 comparing the characteristics of 131 community-associated MRSA (CA-MRSA) cases with 937 hospital-associated MRSA (HA-MRSA) cases in Minnesota, found that CA-MRSA cases had a significantly lower median age, were more likely to be non-white, and had lower average household income than HA-MRSA cases [49]. Additionally, clinical indication of skin and soft tissue infection was more common among CA-MRSA cases, while respiratory and urinary tract infections were more common among HA-MRSA cases. Individuals outside of healthcare settings may comprise an important population at risk for developing *S. aureus* disease, and efforts to prevent the progression from colonization to disease in the community may help to reduce the incidence of serious disease that requires hospitalization. Addressing the issue of community-associated *S. aureus* infections and the progression from colonization to disease may also require consideration of high-risk settings for infection and transmission [50–52], as well as the ethnic and socioeconomic distributions of participants [49, 53, 54].

## Conclusion

More rigorous investigation is needed to accurately quantify the risk of progression to disease after colonization due to a shortage of studies on this subject and the observed heterogeneity between studies. While this variance between studies is expected in any pooled analysis, major sources of variance could be mitigated with some aspects of the study design such as standardization of measurements for colonization and laboratory confirmation of disease. Nevertheless, in the absence of classic hospital-associated risk factors for symptomatic infection, colonization was a significant risk factor for disease for both MRSA and *S. aureus* overall. This finding both supports prior observations that colonization is a risk factor for disease and emphasizes the need for more studies addressing the topic of community-associated *S. aureus* colonization and disease.

## Additional files

Additional file 1: Literature Review / Full-Text Review. The “Literature Review” section contains literature search history, key words used, number of journal articles yielded in each search, and the number of journal articles that have passed each step of the selection process according to the inclusion and exclusion criteria. The “Full-Text Review” section contains all of the information gathered from the selected journal article as it pertained to inclusion into the meta-analysis. (XLS 67 kb)

Additional file 2: Characteristics of studies selected for inclusion in meta-analysis. This file contains descriptive information on the studies included in the meta-analysis: authors, study design, location, study period, number of subjects, study population, study setting, study subjects, disease types, and *S. aureus* resistance subgroup. (PDF 420 kb)

Additional file 3: Funnel Plot – All *S. aureus*. This funnel plot represents an assessment for the presence of publication bias in studies reporting data on colonization and disease associated with all *S. aureus*. This figure compares the effect size (log(OR)) to variance (the standard error of log(OR)) for each individual study. However, because there are fewer than 10 studies featured in this example, we cannot reliably distinguish between chance and true publication bias. (PDF 17 kb)

Additional file 4: Funnel Plot – MRSA. This funnel plot represents an assessment for the presence of publication bias in studies reporting data on colonization and disease associated with MRSA. This figure compares the effect size (log(OR)) to variance (the standard error of log(OR)) for each individual study. However, because there are fewer than 10 studies featured in this example, we cannot reliably distinguish between chance and true publication bias. (PDF 17 kb)

Additional file 5: Funnel Plot – MSSA. This funnel plot represents an assessment for the presence of publication bias in studies reporting data on colonization and disease associated with MSSA. This figure compares the effect size (log(OR)) to variance (the standard error of log(OR)) for each individual study. However, because there are fewer than 10 studies featured in this example, we cannot reliably distinguish between chance and true publication bias. (PDF 16 kb)

## Abbreviations

CA-MRSA: Community-associated MRSA
HA-MRSA: Hospital-associated MRSA
HIV: Human immunodeficiency virus
MRSA: Methicillin-resistant *Staphylococcus aureus*
MSSA: Methicillin-sensitive *Staphylococcus aureus*
OR: Odds ratio
PCR: Polymerase chain reaction
PRISMA: Preferred Reporting Items for Systematic Reviews and Meta-Analyses
*S. aureus*: *Staphylococcus aureus*
SSTI: Skin and soft-tissue infection
