# Supplementary material for: The association between community-associated Staphylococcus aureus colonization and disease: a meta-analysis
Source: BMC Infect Dis. 2018 Feb 21;18:86. doi: 10.1186/s12879-018-2990-3 (PMC5822478; doi:10.1186/s12879-018-2990-3)
Supplement: Supplementary file 2 — Characteristics of studies selected for inclusion in meta-analysis. This file contains descriptive information on the studies included in the meta-analysis: authors, study design, location, study period, number of subjects, study population, study setting, study subjects, disease types, and S. aureus resistance subgroup. (PDF 420 kb) [file 12879_2018_2990_MOESM2_ESM.pdf]

Additional file 2 – Characteristics of studies selected for inclusion in meta-analysis

Article	Study design	Location(s)	Study period	Study subjects (N)	Study Population	Study Setting	Disease type(s) reported	*S. aureus* type subgroup*
Nguyen et al., 1999[32]	Prospective cohort	USA	Not reported	201	HIV-infected outpatients (adults)	Outpatient clinic at Veterans Affairs Medical Centers (Michigan, Massachusetts, Pennsylvania)	SSTI, bacteremia, endocarditis, pneumonia	SA
Gordon et al., 2005[30]	Prospective cohort	USA	1/2001-12/2001	75	HIV-infected patients with a history of drug use participating in Project Samaritan (adults)	Residential drug treatment facility (Bronx, NY)	SSTI, bacteremia	SA
Ellis et al., 2009[21]	Prospective cohort	USA	1/2005-12/2005	3066	U.S. Army personnel training to be combat medics (adults)	U.S. army post Fort Sam Houston (San Antonio, TX)	SSTI	MRSA
Fritz et al., 2009[24]	Prospective cohort	USA	10/2005-6/2006	534	Pediatric outpatients at well and sick visits (children age 0-18)	Catchment area of Washington University Pediatric and Adolescent Ambulatory Research Consortium (WA)	SSTI	SA, MRSA, MSSA
Miller et al., 2009[22]	Cross-sectional	USA	2/2004-6/2006	914	Community households, selected by random digit dialing (adults and children >1 year of age)	Catchment area of Columbia University Medical Center (Manhattan, NY)	SSTI	SA
Shet et al., 2009[29]	Prospective cohort	USA	2005-2006	107	HIV-1 infected MSM outpatients w/ no evidence of immune suppression (adults)	Outpatient clinic of Aaron Diamond AIDS Research Center Clinical Program at Rockefeller Hospital (New York, NY)	SSTI	MRSA
Szumowski et al., 2009[23]	Prospective cohort	USA	10/2005-1/2007	795	Outpatients (adults); 243/795 HIV-infected	Outpatient clinics of Fenway Community Health (Boston, MA)	SSTI	MRSA
Lo et al., 2010[28]	Cross-sectional	Taiwan	2005-2006	153	Pediatric outpatients with atopic dermatitis (<= 14 years old)	Pediatric department of Tri-Service General Hospital (Taipei)	SSTI	SA, MRSA, MSSA
Maree et al., 2010[27]	Case-control	USA	10/2006-1/2007	162	Male prison inmates (adults)	County Sheriff's Department jail facilities (Los Angeles, CA)	SSTI	SA, MRSA, MSSA
Oliva et al., 2013[31]	Cross-sectional	Italy	1/2008-6/2008	63	HIV-infected outpatients (adults)	Outpatient clinic at the Department of Infectious and Tropical Diseases of Sapienza University of Rome (Rome)	SSTI	MSSA
Peters et al., 2013[25]	Prospective cohort	USA	9/2007-4/2008	600	HIV-infected, 53% MSM (adults > 18 years of age)	Veterans Affairs Medical Center HIV Clinic (Atlanta, GA)	SSTI, bacteremia, pneumonia	MRSA
Miko et al., 2015[26]	Case-control	USA	3/2011-1/2013	328	Prison inmates (>= 16 years of age)	Maximum security prisons: Sing Sing Correctional Facility, Bedford Hills Correctional Facility (NY)	SSTI	SA

SSTI = skin and soft tissue infection, MSM = men who have sex with men

* MRSA = methicillin-resistant *Staphylococcus aureus*, MSSA = methicillin-susceptible *S. aureus*, SA = *S. aureus*
