# Supplementary figures and images for: The association between community-associated Staphylococcus aureus colonization and disease: a meta-analysis
Source: BMC Infect Dis. 2018 Feb 21;18:86. doi: 10.1186/s12879-018-2990-3 (PMC5822478; doi:10.1186/s12879-018-2990-3)

Funnel plot with pseudo 95% confidence limits

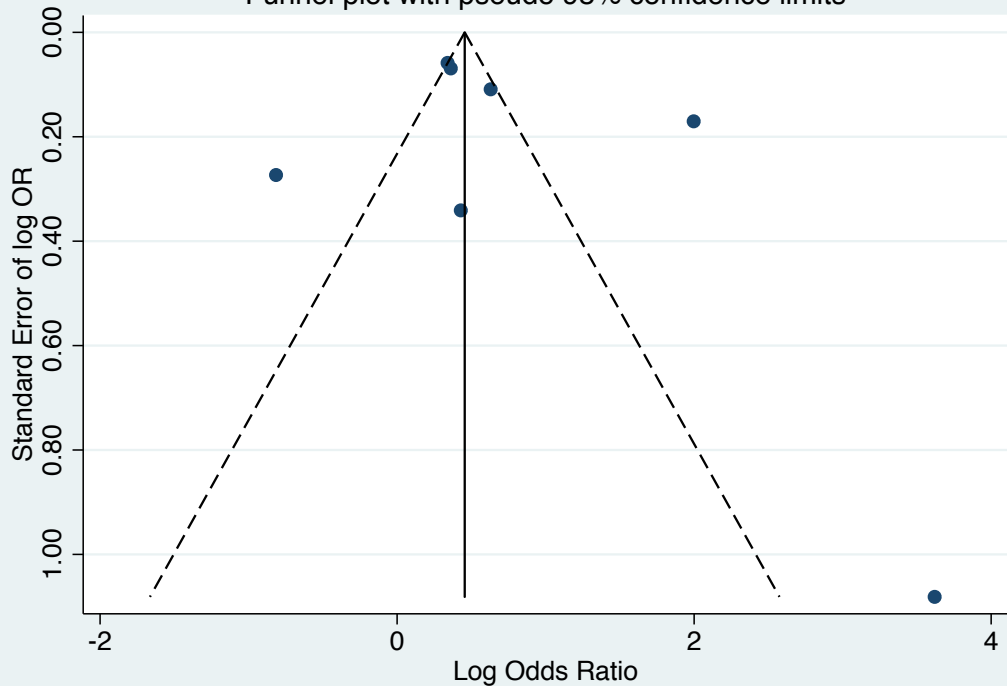

Supplement: Supplementary file 3 — Funnel Plot – All S. aureus. This funnel plot represents an assessment for the presence of publication bias in studies reporting data on colonization and disease associated with all S. aureus. This figure compares the effect size (log(OR)) to variance (the standard error of log(OR)) for each individual study. However, because there are fewer than 10 studies featured in this example, we cannot reliably distinguish between chance and true publication bias. (PDF 17 kb) [file 12879_2018_2990_MOESM3_ESM.pdf]

Funnel plot with pseudo 95% confidence limits

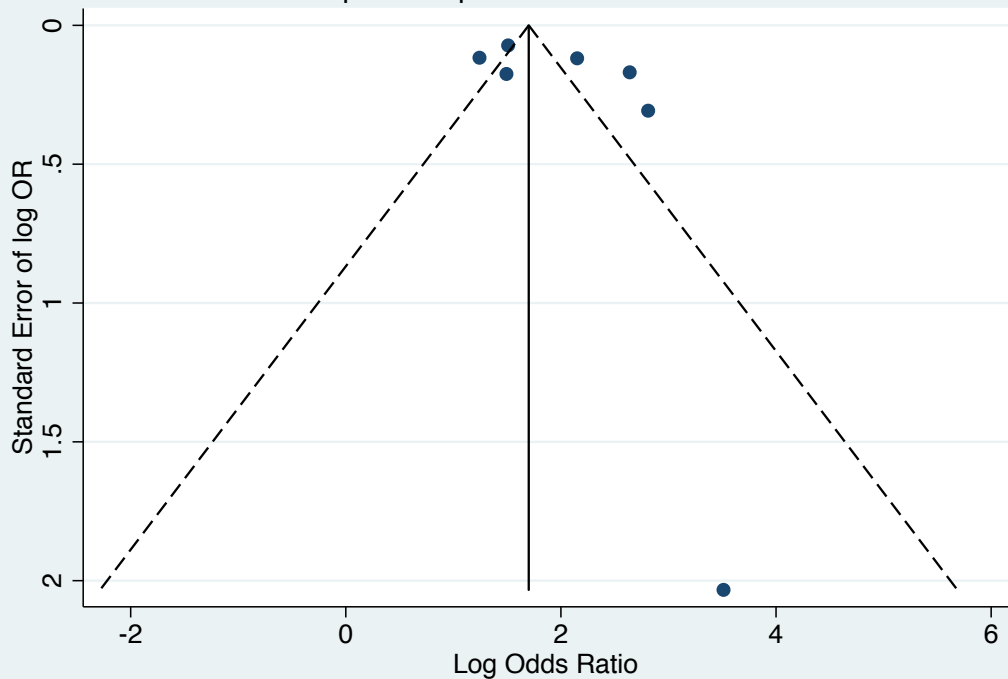

Supplement: Supplementary file 4 — Funnel Plot – MRSA. This funnel plot represents an assessment for the presence of publication bias in studies reporting data on colonization and disease associated with MRSA. This figure compares the effect size (log(OR)) to variance (the standard error of log(OR)) for each individual study. However, because there are fewer than 10 studies featured in this example, we cannot reliably distinguish between chance and true publication bias. (PDF 17 kb) [file 12879_2018_2990_MOESM4_ESM.pdf]

Funnel plot with pseudo 95% confidence limits

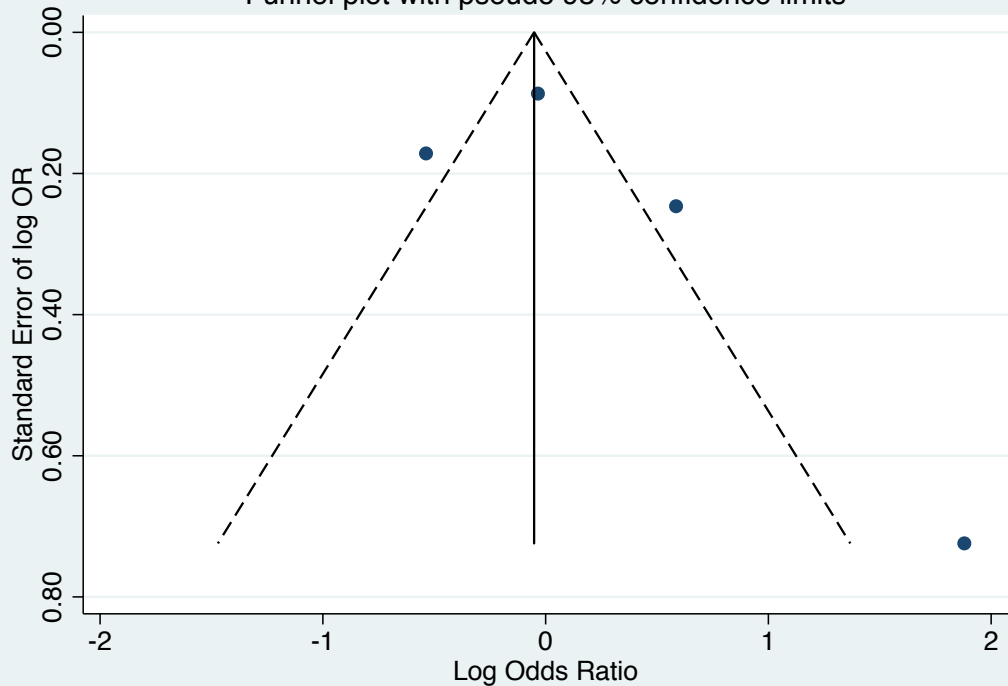

Supplement: Supplementary file 5 — Funnel Plot – MSSA. This funnel plot represents an assessment for the presence of publication bias in studies reporting data on colonization and disease associated with MSSA. This figure compares the effect size (log(OR)) to variance (the standard error of log(OR)) for each individual study. However, because there are fewer than 10 studies featured in this example, we cannot reliably distinguish between chance and true publication bias. (PDF 16 kb) [file 12879_2018_2990_MOESM5_ESM.pdf]
